# Hydroprocessing of Jatropha Oil for Production of Green Diesel over Non-sulfided Ni-PTA/Al_2_O_3_ Catalyst

**DOI:** 10.1038/srep11327

**Published:** 2015-07-10

**Authors:** Jing Liu, Jiandu Lei, Jing He, Lihong Deng, Luying Wang, Kai Fan, Long Rong

**Affiliations:** 1 MOE Key Laboratory of Wooden Material Science and Application, Beijing Forestry University, Beijing 100083, P. R. China; 2 Key Laboratory for Biomechanics and Mechanobiology of Ministry of Education, School of Biological Science and Medical Engineering, Beihang University, Beijing 100191, P. R. China

## Abstract

The non-sulfided Ni-PTA/Al_2_O_3_ catalyst was developed to produce green diesel from the hydroprocessing of Jatropha oil. The Ni-PTA/Al_2_O_3_ catalyst was prepared by one-pot synthesis of Ni/Al_2_O_3_ with the co-precipitation method and then impregnanting Ni/Al_2_O_3_ with PTA solution. The catalysts were characterized with BET, SEM-EDX, TEM, XRD, XPS, TGA and NH_3_-TPD. The Ni and W species of the Ni-PTA/Al_2_O_3_ catalyst were much more homogeneously distributed on the surface than that of commercial Al_2_O_3_. Catalytic performance in the hydroprocessing of Jatropha oil was evaluated by GC. The maximum conversion of Jatropha oil (98.5 wt%) and selectivity of the C15-C18 alkanes fraction (84.5 wt %) occurred at 360 °C, 3.0 MPa, 0.8 h^−1^. The non-sulfided Ni-PTA/Al_2_O_3_ catalyst is more environmentally friendly than the conventional sulfided hydroprocessing catalyst, and it exhibited the highest catalytic activity than the Ni-PTA catalyst supported with commercial Al_2_O_3_ grain and Al_2_O_3_ powder.

Renewable and clean fuel sources are currently in high demand, due to environmental challenges such as climate change, diminishing fossil fuel reserves and deteriorating quality of crude oil^1^. In recent years, hydroprocessing of vegetable oil for hydrocarbons (commonly called green diesel) has become more popular. Jatropha oil has several advantages. It is an ideal source of triglycerides does not compete with arable land for food, and may yield more biofuel per hectare than canola, sunflower, and soyabeans^2^. Its hydroprocessing product contains mainly normal C17 and C18 paraffins with a high cetane number.

In the past, conventional hydrogenation catalysts such as sulfided NiMo-alumina or CoMo-alumina have been commonly used^3^, and noble metal catalysts such as Pt or Pd based-catalysts have been also reported^4^. Alumina is popular due to its moderate acidity and reduced cracking activity, leading to a high yield of green diesel. Normal alumina has a relatively large specific surface area (about 200 m^2^/g), but still cannot meet the demand of the chemical and petrochemical industries. A catalyst with a small pore diameter or low specific surface area would decrease its catalytic activity by restricting the access of reactants to the catalytic sites or decreasing the numbers of activity sites per unit area^5^. Appropriate amount of macropores can promote the desorption of the products, following by the inhibition of some side reactions^6^. Yin *et al.*^7^ studied the effect of alumina support on the catalytic performance of Pt-Sn/Al_2_O_3_ catalysts in one-step synthesis of N-phenylbenzylamine from aniline and benzyl alcohol. They found that the large pore volumes and pore size distributions of alumina supports aided in diffusion and adsorption of reactants on the catalyst surface and increased the catalytic activity. Alumina supports were also found to help remove products from the catalyst surface and enhanced catalytic stability.

The use of alumina-supported transition metal as a catalyst has been found^8^,^9^,^10^ to produce n-alkanes from triglycerides of palm oil, soybean oil and other vegetable oils. However, few studies have explored the effects of of alumina structure on hydroprocessing activity. Furthermore, transition metal catalysts are usually sulfided to retain their active form^11^. This may cause sulfur dioxide emissions, corrosion and sulfur residues in the products under long-term reaction, since vegetable oils are free of sulfur compounds. Thus the quality of green diesel might be affected if the product oil contained sulfur residuals. Although noble metal catalysts showed high catalytic activity without sulfidizing process, they were not suitable for the large-scale process due to their high cost. Our previous work successfully prepared non-sulfided rare metal^12^ and heteropolyacid catalysts^13^,^14^ prepared by wet co-impregnation method to produce the straight chain alkanes C15 to C18. In the current study, we prepared alumina-supported nickel and phosphotungstic acid (Ni-PTA/Al_2_O_3_) catalysts with a large pore volume and pore size to produce green diesel without sulfidation. Large pore sizes favor the diffusion of large-size fatty acid molecules^15^. The co-precipitation method was used to prepare Ni/Al_2_O_3_ in order to obtain homogenous and more metal activity sites. In addition, we compared the hydroprocessing activity of catalysts with synthetic alumina support to that of commercial alumina supports.

## Results

### Catalyst characterization

**Figure 1:**
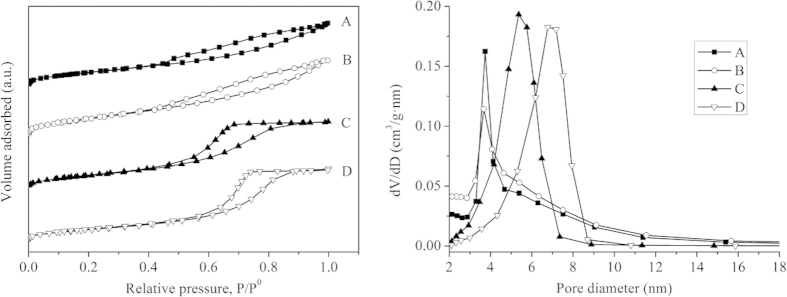
Nitrogen adsorption-desorption isotherms and pore size distribution of (**A**) commercial Al_2_O_3_ grain, (**B**) commercial Al_2_O_3_ powder, (**C**) synthetic Al_2_O_3_ support and (**D**) synthetic Ni-PTA/Al_2_O_3_ catalyst.

The nitrogen adsorption-desorption isotherms of the Al_2_O_3_ samples were showed in Fig. 1. The adsorption of two commercial alumina supports (A: Al_2_O_3_ grain and B: Al_2_O_3_ powder) increased gradually as relative pressure increased, and then increased rapidly at a high relative pressure (P/P^0^ > 0.4). This displayed Langmuir type IV isotherms with a H4-type hysteresis loop in the IUPAC classification^16^. This type is often associated with a typical mesoporous material with size-homogeneous 1D slit channels^17^. The synthetic alumina supports (C) and Ni-PTA/Al_2_O_3_ catalyst (D) exhibited H2-type hysteresis loops characteristic of solids with ink bottle pores. The pore network structure may be interconnected or independent^18^. According to the nitrogen adsorption-desorption isotherms, it is evident that the total adsorption of the synthetic samples was larger than that of the commercial samples.

Pore size distribution can be calculated from the desorption branches of the isotherms according to the BJH (Barrett-Joyner-Halenda) method. Curves were displayed in Fig. 1. Each sample presented a characteristic of mesoporous structure. A comparison of the textural properties of the Al_2_O_3_ samples was shown in Table 1. Commercial Al_2_O_3_ grain (A) and Al_2_O_3_ powder (B) both have a very narrow pore size distribution, with an average pore diameter of 5.18 nm and 4.84 nm, respectively. In synthetic alumina supports (C) and Ni-PTA/Al_2_O_3_ catalyst (D), the curves presented a unimodal pore structure with a little wider distribution in the mesoporous range (2–7 nm and 2–9 nm, respectively). Table 1 shows that synthetic Al_2_O_3_ support had a considerably high surface area (296 m^2^/g), while the surface area of the prepared catalyst was lower than that of the Al_2_O_3_ support due to the deposit of the metal on the support surface^19^.

**Figure 2:**
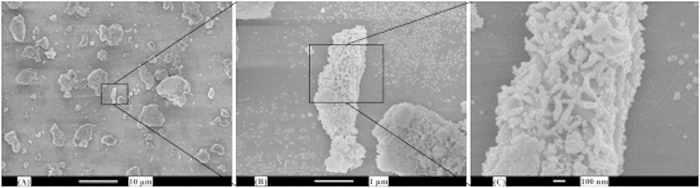
Scanning electron microscope images of synthetic Al_2_O_3_ support (**A**) × 2000, (**B**) × 20000 and (**C**) × 60000.

The synthetic Al_2_O_3_ support was characterized by SEM, as shown in Fig. 2. Scanning electron micrographs showed the morphologies and pore structure. It is evident that the synthetic Al_2_O_3_ support presented a mesoporous structure with small pores. The pore structure was identical with the results obtained from BET.

**Figure 3:**
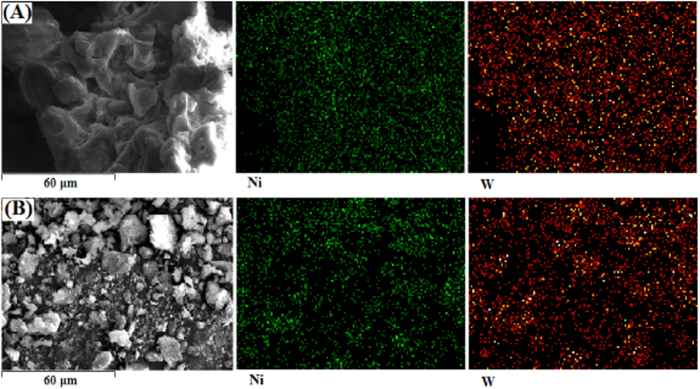
SEM and EDX elemental mappings for the synthetic Ni-PTA/Al_2_O_3_ catalyst (A) and Ni-PTA/Al_2_O_3_ powder (B).

Figure 3 showed the Ni and W species distributed on the alumina surface as shown in SEM-EDX images of synthetic Ni-PTA/Al_2_O_3_ catalyst and Ni-PTA/Al_2_O_3_ powder. The Ni and W species on the synthetic Ni-PTA/Al_2_O_3_ catalyst (Fig. 3A) were much more homogeneously distributed on the surface. However, Ni and W on the Ni-PTA/Al_2_O_3_ powder (Fig. 3B) displayed large spots, suggesting that the Ni and W were not well distributed compared with synthetic Ni-PTA/Al_2_O_3_ catalyst. Furthermore, EDX measurements were made to determine the chemical composition of the catalyst, and quantitative analysis of different elements showed that the synthetic Ni-PTA/Al_2_O_3_ catalyst (A) contained 5.74 wt % Ni, 11.63 wt % W, 0.80 wt % P and 35.09 wt % Al, while Ni-PTA/Al_2_O_3_ powder (B) contained 2.43 wt % Ni, 7.93 wt % W, 0.23 wt % P and 34.05 wt % Al. This indicated that the metal content of synthetic Ni-PTA/Al_2_O_3_ catalyst was higher than that of the Ni-PTA/Al_2_O_3_ powder.

**Figure 4:**
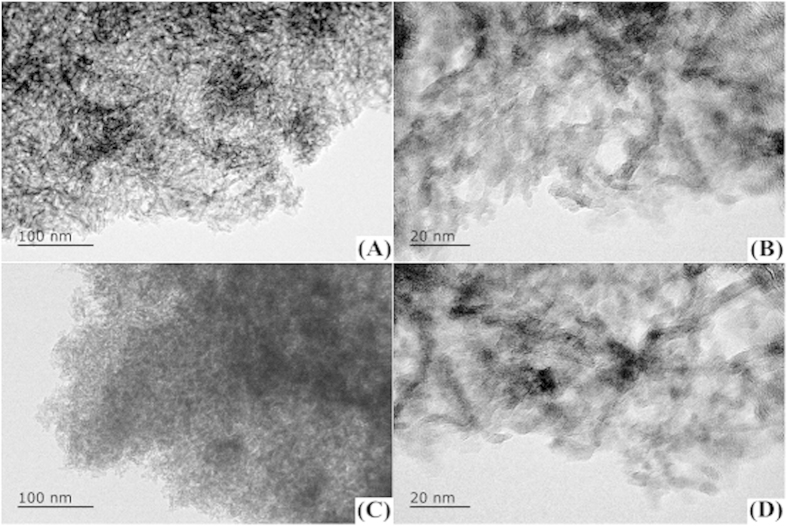
Transmission electron microscopy images of the synthetic Ni/Al_2_O_3_ catalyst (**A**) ×200k, (**B**) ×800k and synthetic Ni-PTA/Al_2_O_3_ catalyst (**C**) ×200k, (**D**) ×800k.

The transmission electron microscopy technique was used to further measure the nickel particle size and dispersion of metal. TEM images of synthetic Ni/Al_2_O_3_ and Ni-PTA/Al_2_O_3_ catalysts were shown in Fig. 4. The black nickel particles were clearly evident in the images, and the dimension of nickel particle estimated from the TEM image was about 10 nm. Figure 4A,B showed the composition of random, stack-like particles. The distribution of Ni particles was relatively uniform in Fig. 4C,D. This suggested that adding PTA may promote the dispersion of Ni particles. Moreover, the pore diameter from Fig. 4B,D was about 2–10 nm, which was confirmed by BET results. It can be deduced that the mesopores were formed due to a slew of particles connected mutually during co-precipitation and the intervals among particles formed during calcination.

**Figure 5:**
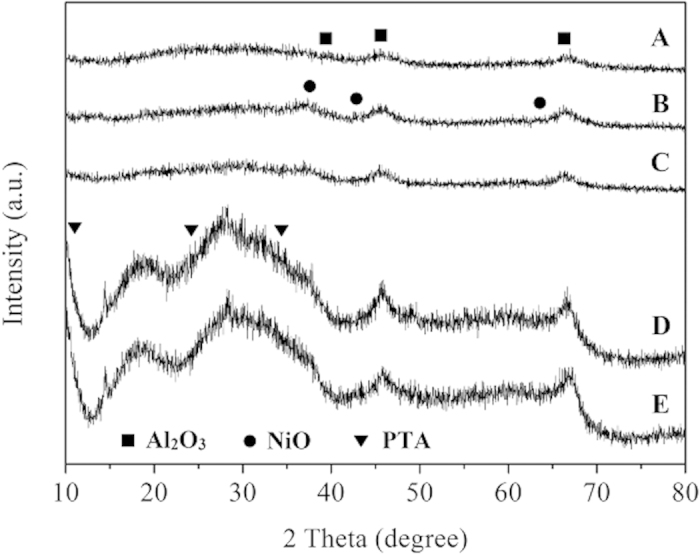
XRD patterns of (**A**) synthetic Al_2_O_3_ support, (**B**) synthetic Ni/Al_2_O_3_ catalyst and (**C**) synthetic Ni-PTA/Al_2_O_3_ catalyst, (**D**) Ni-PTA/Al_2_O_3_ grain, (**E**) Ni-PTA/Al_2_O_3_ powder.

The XRD pattern of synthetic Al_2_O_3_ support, Ni/Al_2_O_3_ catalyst and Ni-PTA/Al_2_O_3_ catalyst were shown in Fig. 5. All samples exhibited characteristic peaks at 2θ = 38°, 46° and 66° correspond to (311), (400) and (440) plane of γ-Al_2_O_3_, respectively^5^. The peaks observed at 2θ = 37°, 43°, and 63° were attributed to the Ni oxide phase (JCPDS No. 4–835). NiO and Ni_2_O_3_ were the most common Ni oxides, and may also exist in the microcrystalline or amorphous phases, while NiAl_2_O_4_ peaks may overlap with the peaks of γ-Al_2_O_3_^8^. The PTA phase (11°, 26°, and 34°, JCPDS No. 50–0657) was not observed, as shown in Fig. 5C. This indicated that the PTA was highly dispersed on the catalyst surface, forming an amorphous surface compound. In addition, there was no diffraction peak in the synthetic Ni-PTA/Al_2_O_3_ catalyst sample, indicating that the Keggin structure remains intact^20^. However, Ni-PTA/Al_2_O_3_ grain and powder catalysts (Fig. 5D,E) exhibited diffraction peaks for PTA. This implied that the crystalline structure of PTA was kept in the mixture, and that the PTA was aggregated due to excessive impregnation onto Al_2_O_3_. This accords with SEM-EDX results (Fig. 3), which showed the differentiated distribution of PTA (Ni and W) on the Ni-PTA/Al_2_O_3_ powder.

The XRD patterns of synthetic Ni-PTA/Al_2_O_3_ catalyst with PTA loading (30%) showed only the diffraction pattern of NiO and Al_2_O_3_, and no diffraction peak due to PTA was observed. These results suggested that the PTA particles dispersed well on the surface of NiO-doped samples. Compared with the Ni/Al_2_O_3_ catalyst (B), the peaks of the Ni-PTA/Al_2_O_3_ catalyst (C) were weaker, indicating that the added PTA could increase the dispersion of Ni, leading to the formation of a more active center and increasing catalytic activity.

In addition, TG-DTA analysis (see Supplementary data of Figure S1) confirmed that there was a small loss of weight (3.97%) in the TG curve of PTA (H_3_PW_12_O_40_·6H_2_O), and two separated peaks deriving from dehydration of physically adsorbed and crystal water (the loss of 6H_2_O molecules) were observed at temperatures below 125 °C and around 150–325 °C in DTA curve. From the DTA curves, it was found that PTA showed one obvious endothermic peak at around 225 °C, and another exothermic peak appeared at 610 °C which is due to the crystallization of tungstate species formed by the decomposition of PTA. These results were consistent with the reported literature^21^,^22^. From the analysis of TG-DTA, it can be concluded that PTA species in the catalyst remain its Keggin structure after calcination at 400 °C in the hydroprocessing, and the observed cracking activity was due to PTA.

**Figure 6:**
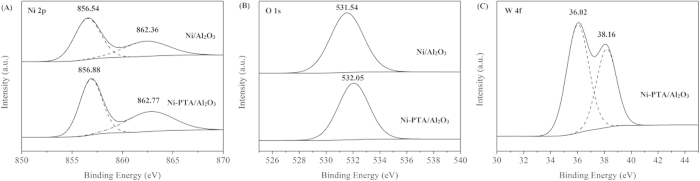
XPS spectra of (**a**) Ni 2p, (**b**) O 1s and (**c**) W 4f levels of the synthetic Ni/Al_2_O_3_ catalyst and the synthetic Ni-PTA/Al_2_O_3_ catalyst.

XPS spectra for the synthetic Ni/Al_2_O_3_ catalyst and the synthetic Ni-PTA/Al_2_O_3_ catalyst were shown in Fig. 6. The Ni 2p binding energies at 856–863 eV were observed in Fig. 6A for the two catalysts. As expected, nickel is present in the Ni^2+^ oxidation state on the surface. The binding energy of Ni 2p in the region near 856.54 eV is attributed to Ni^2+^ ions, whereas that around 862.36 eV is known as a satellite peak that appears as a result of multielectron excitation^23^. The shift of the peak towards higher binding energy values for Ni-PTA/Al_2_O_3_ is assigned to the stronger interaction between Ni^2+^ and the alumina surface^24^.

Figure 6B showed the O 1s XPS spectra for the two catalysts. The O 1s peak position shifts towards higher binding energy values for the Ni-PTA/Al_2_O_3_ catalyst with a stronger acidic character. The shift towards higher binding energy of more than 0.5 eV for the O 1s orbital passing from Ni/Al_2_O_3_ to Ni-PTA/Al_2_O_3_ is associated with a strong electronegative effect of the chemical species bonded to the acidic surface oxygen sites. This attracts the electron clouds of the Ni-PTA/Al_2_O_3_, and increased the bond polarization with terminal hydrogen atoms^25^. Thus, the O 1s peak shift towards high binding energy is indicative of a more acidic behavior and the peak of Ni-PTA/Al_2_O_3_ catalyst is probably corresponding to W-O-H^26^.

As reported by P.A. Jalil *et al.*^27^, the W 4f region in pure PTA spectrum showed the spin-orbit split doublet 4f_7/2_ and 4f_5/2_ located at 35.8 and 38.0 eV, respectively. Similarly, as shown in Fig. 6C, the Ni-PTA/Al_2_O_3_ catalyst exhibited the spin-orbit splitting signals of W4f_7/2_ and W4f_5/2_ at binding energy of 36.02 and 38.16 eV, respectively. This result clearly showed the existence of assembled PTA on Ni/Al_2_O_3_ catalyst. The peaks at low energy were attributed to W^x+^(W^x+^represents nonstoichiometric WO_x_/W) and the peaks at high energy were attributed to W^6+^, in agreement with our previous report^28^.

In addition, from the XPS spectra of the Ni 2p level and W 4f level in Fig. 6A and Fig. 6C, respectively, we can see that Ni and W were present in the form of oxides or hydroxides, which acted as spacers. These metal oxide/hydroxides not only protected the amorphous structure of the catalyst, aiding in hydrogenation activity, but also enhanced the dispersion of active sites^29^. Combining the results of SEM-EDX and XRD, it is reasonable to point out that the metal active sites should be highly dispersed, leading to the formation of more active centers then markedly increasing the catalytic activity.

**Figure 7:**
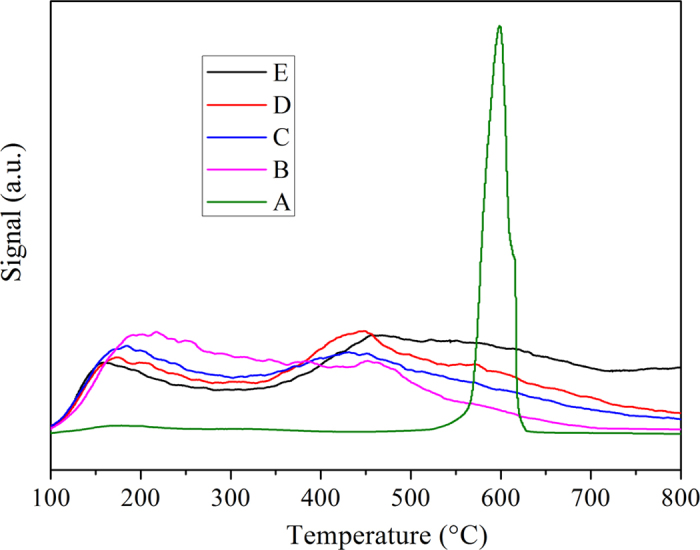
NH_3_-TPD profiles of (**A**) pure PTA, (**B**) synthetic Al_2_O_3_ support, (**C**) Ni-PTA(10)/Al_2_O_3_ catalyst, (**D**) Ni-PTA(30)/Al_2_O_3_ catalyst and (**E**) Ni-PTA(50)/Al_2_O_3_ catalyst.

NH_3_-TPD was carried out to evaluate the acidic properties of the samples. NH_3_-TPD profiles of pure PTA, synthetic Al_2_O_3_ support and catalysts with different PTA loadings were shown in Fig. 7. The adsorbed NH_3_ molecules desorbed from weak acid sites at low temperatures, and from strong acid sites at high temperatures. The TPD profile of pure PTA (Fig. 7A) presented only one desorption peak (maximum 600 °C) in the temperature range of 100–800 °C, while synthetic Al_2_O_3_ support (Fig. 7B) showed a broad distribution of the desorption peak in the temperature range of 100–800 °C, with a peak maxima at 217 °C. From the NH_3_-TPD profiles of Ni-PTA(10–50)/Al_2_O_3_ catalyst (Fig. 7C–E), it was found that all catalysts showed similar acid site distribution, but differences in concentration. The Ni-PTA(10–50)/Al_2_O_3_ catalyst showed two desorption peaks in the temperature range of 100–300 °C and 300–800 °C, and had two types of acid sites (weak acid sites and strong acid sites) on the surfaces. However, pure PTA did not show an obvious peak in the temperature range of 100–300 °C, while synthetic Al_2_O_3_ support (Fig. 7B) did not show an obvious peak in the temperature range of 300–800 °C, indicating that PTA only had strong acid sites and Al_2_O_3_ did not have strong acid sites on the surface. It was evident from Fig. 7C–E that weak acid sites of all catalyst surfaces decreased with the addition of PTA from 10–50%, while a monotonic increase in the numbers of strong acid sites as the PTA loading rose. The nature of a solid acid catalyst was mainly determined by the strongest acid sites on the surface^30^. Compared with desorption peak of pure PTA (Fig. 7A), that assigned to the strong acid sites were shifted to lower temperature (about 450 °C), showing that the strength of acid sites were further receded, which strongly weaken the coke deposition and restrain the deactivation of the catalyst.

All tested samples showed similar acid site distribution but differences in concentration. Desorption temperatures and the amount of ammonia desorbed on each of the TPD peaks were shown in Table 2. Pure PTA, synthetic support and catalysts all had considerable acidity. The relative ratio of weak acidities decreased in the order: Synthetic Al_2_O_3_ > Ni-PTA(10) > Ni-PTA(30) ≈ Ni-PTA(50) >> PTA. From these data, it is evident that strong acidity of Al_2_O_3_ surface increased with the PTA loading up to 30 wt.%, and then decreased. It is suggested that the much higher loading leads to the PTA units not spreading well on the Al_2_O_3_ surface and their serious agglomeration, thus lowering the number of accessible strong acid sites^31^.

**Figure 8:**
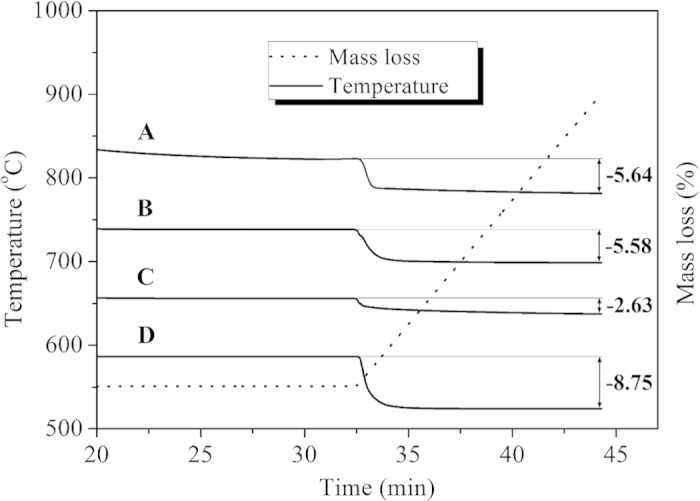
TGA profiles of (**A**) Ni-PTA/Al_2_O_3_ grain after use for 10 h, (**B**) Ni-PTA/Al_2_O_3_ powder after use for 10 h, (**C**) synthetic Ni-PTA/Al_2_O_3_ catalyst after use for 10 h and (**D**) 70 h. Reaction conditions: 360 °C, 3.0 MPa, 0.8 h^−1^.

The coke content in used catalysts was quantitatively analyzed by TGA measurements, and the curves were shown in Fig. 8. According to the reference of ISO 6964-1986, the decoking of carbon deposited on the catalyst surface usually occurred at over 550 °C in an oxidative environment^32^. As shown in Fig. 8, the amounts of carbon deposited on the Ni-PTA/Al_2_O_3_ grain (A = 5.64%) and Ni-PTA/Al_2_O_3_ powder (B = 5.58%) were both higher than that on synthetic Ni-PTA/Al_2_O_3_ catalyst (C = 2.63%). Compared the reaction of 10 h, the carbon residue of NiMoLa/Al_2_O_3_ catalyst (D = 8.75%) after the reaction of 70 h increased 6.12%. These results confirmed that the total amount of coke produced varied with different types of support, and the amount of coke on synthetic Ni-PTA/Al_2_O_3_ catalyst after use for 10 h was lowest. Since the reactivity of coke depends on the structure of the catalysts, which was closely related to the morphology of the catalyst^33^, the synthetic catalyst showed a different textural property and morphology than other commercial catalysts studied. This result is in accordance with the BET results, which showed that the surface area and pore diameter of synthetic Al_2_O_3_ support was higher than that of the commercial samples.

### Effect of reaction temperature on the composition of product oil

**Figure 9:**
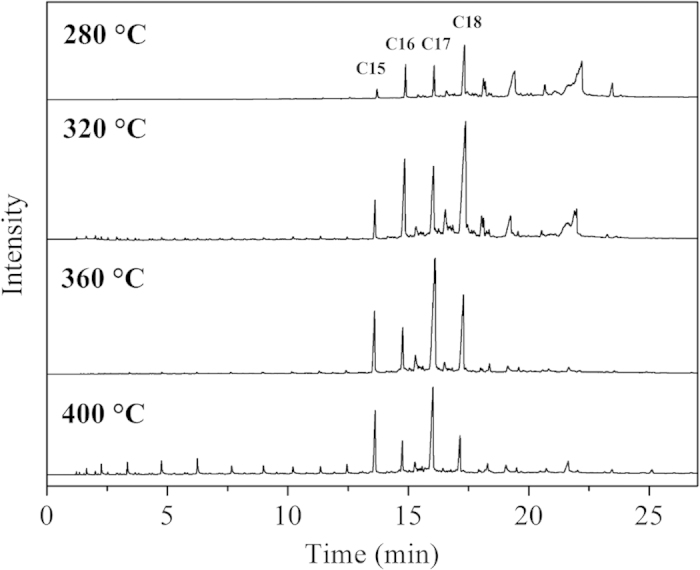
GC charts of product oil from hydroprocessing of Jatropha oil over synthetic Ni-PTA(10)/Al_2_O_3_ catalyst at 280–400 °C, 3.0 MPa, 0.8 h^−1^.

Hydroprocessing temperature is a key parameter for catalyst performance. Figure 9 showed GC charts of liquid product oil from hydroprocessing of Jatropha oil over a synthetic Ni-PTA(10)/Al_2_O_3_ catalyst on 280–400 °C. As shown in Fig. 9, although C15H32, C16H34, C17H36 and C18H38 were the main products at different temperatures, the amount of smaller molecule alkane (<C15) that was formed increased remarkably when the reaction temperature reached 400 °C. When the reaction was carried out at a low reaction temperature of 280 and 320 °C, the peaks of intermediates (such as fatty acids or fatty esters)^34^ were observed in the GC chart. At temperatures higher than about 360 °C, intermediates were not detected in the product oil. On the other hand, reaction temperature had a relatively strong influence on the ratio of n-alkanes with an even and odd carbon atom number^35^. It was apparent that as the reaction temperature increased, the content of n-C17 increased at the expense of n-C18.

**Figure 10:**
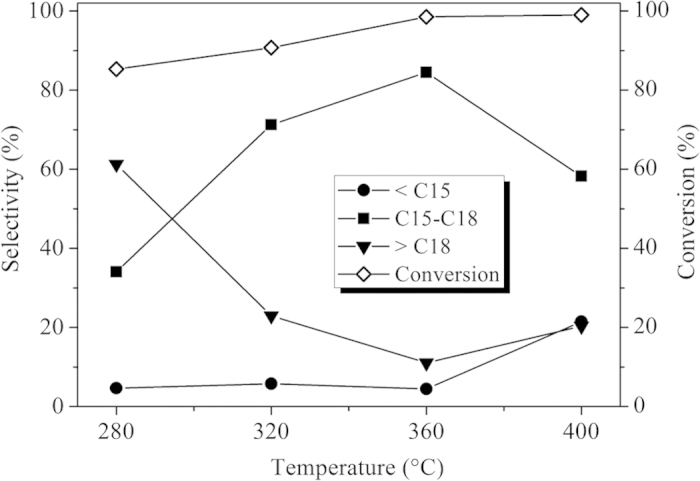
Effect of reaction temperature on selectivity of product oil and conversion of Jatropha oil using a synthetic Ni-PTA(10)/Al_2_O_3_ catalyst.

Jatropha oil was hydroprocessed at a reaction temperatures ranging from 280 to 400 °C at 3.0 MPa, 0.8 h^−1^. Results for selectivity of product oil and conversion of Jatropha oil were presented in Fig. 10. Conversion increased with increasing reaction temperature, and the lowest conversion exceeded 85% at the lowest temperature (280 °C). The C15-C18 selectivity increased from 34.1 to 84.5% as the reaction temperature increased from 280 to 360 °C. This increase is due to an increase in the conversion of triglycerides, free fatty acids and oxygenated intermediates to alkanes^36^. This is evident in the decreasing selectivity trend of the >C18 fraction, as shown in Fig. 10. The maximum selectivity of the C15-C18 fraction (84.5 wt %) was observed at 360 °C. When the temperature increased to 400 °C, the C15-C18 selectivity significantly decreased and the amount of light hydrocarbons (<C15) greatly increased. This indicated that temperature played an important role in the cracking of the intermediate carbenium ions^4^ at over 360 °C.

### Effect of PTA loading on the composition of product oil

**Figure 11:**
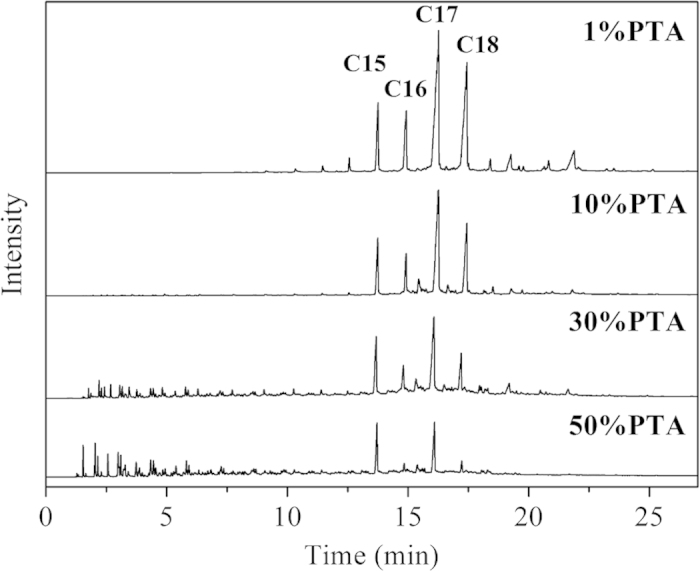
GC charts of product oil from hydroprocessing of Jatropha oil over synthetic Ni-PTA(1–50)/Al_2_O_3_ catalysts at 360 °C, 3.0 MPa, 0.8 h^−1^.

As shown in Fig. 11, the Ni-PTA/Al_2_O_3_ catalyst formed n-C15H32, n-C16H34, n-C17H36, and n-C18H38 as the main products. The concentration of smaller molecule alkane (<C15) and iso-paraffins (iso/n ratio = 0.06, see Table 3) were very low. In order to improve cold flow properties, PTA-content was increased for the isomerization/cracking of C15H32 to C18H38 n-paraffins. Fig. 11 showed the light hydrocarbons (< C15), and iso-paraffins increased with increasing PTA-content. Ni-PTA(50)/Al_2_O_3_ catalysts formed many light hydrocarbons (<C15) and iso-paraffins (iso/n ratio = 1.15, see Table 3). This result accorded with results of NH_3_-TPD (see Fig. 7), which showed that the strong acid sites of all catalysts increase with the addition of PTA from 10–50%. According to the proposed bifunctional mechanism^4^, after C17H36 and C18H38 formed by the hydroprocessing of Jatropha oil on Ni/W sites, the corresponding carbenium intermediates were formed on the acid sites of PTA. The carbenium intermediates underwent cracking to form light hydrocarbons as well as isomerization to form isoparaffins.

The isomerization/cracking of n-paraffins over bifunctional catalysts containing metal and solid acid is very important for improving the properties of liquid hydrocarbon fuels^30^. In Table 3, there was an obvious increase of i-alkane content with increasing PTA-content for all tested catalysts. The liquid hydrocarbons formed over Ni-PTA(10) contained many C15-C18 fractions (78.0 wt%), but the pour point of the product was too high (20 °C) to use as a diesel fuel. Although the pour point of liquid hydrocarbons formed over Ni-PTA(30) and Ni-PTA(50) were low enough, C15-C18 selectivity was also low. This is because it formed a large amount of <C15 fraction (see Fig. 10) on the strong acid sites. Thus, Ni-PTA(10) produced the largest amount of C15-C18 hydrocarbon product (84.5 wt%) with a pour point of −10 °C, which was suitable for producing green diesel from the Jatropha oil.

### Influence of alumina supports on the catalytic activity

**Figure 12:**
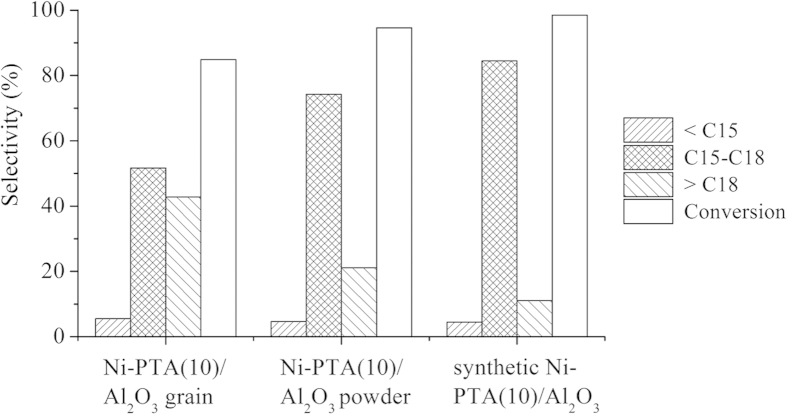
Effect of alumina supports on the selectivity of product oil and conversion of Jatropha oil at 360 °C, 3.0 MPa, 0.8 h^−1^.

Figure 12 showed the conversion of Jatropha oil and product selectivities over Ni-PTA/Al_2_O_3_ grain, Ni-PTA/Al_2_O_3_ powder and synthetic Ni-PTA/Al_2_O_3_ catalysts in the hydroprocessing of Jatropha oil. All PTA content in the catalysts was at 10%. The conversion of Jatropha oil was more than 80% over each catalyst, and increased in the following order: synthetic Ni-PTA/Al_2_O_3_ catalyst > Ni-PTA/Al_2_O_3_ powder > Ni-PTA/Al_2_O_3_ grain. The selectivity of light fraction (<C15) was in a narrow range of 4.5–5.6% over each catalyst. The C15-C18 hydrocarbon selectivity was highest (84.5%) over the synthetic Ni-PTA/Al_2_O_3_ catalyst. It was 51.6% and 74.3% over the Ni-PTA/Al_2_O_3_ grain and Ni-PTA/Al_2_O_3_ powder catalyst, respectively, due to the considerable heavy fraction (>C18). The corresponding XRD data (Fig. 5) revealed that large PTA agglomerates formed in the Ni-PTA/Al_2_O_3_ grain and powder catalysts. These agglomerates cannot enter the pores of the support, and therefore block the pore mouths. Consequently, PTA dispersion and amount of accessible acid sites decreased on the surface of Ni-PTA/Al_2_O_3_ grain and powder catalysts, which led to a decline in the conversion of Jatropha oil and C15-C18 selectivity^37^. From the results of BET and TGA, synthetic Al_2_O_3_ support had the highest surface area (296 m^2^/g), and synthetic Ni-PTA/Al_2_O_3_ catalysts showed the least amount of coke (2.63%). Thus, the results of catalytic activity were consistent with that of BET and TGA. Synthetic Ni-PTA/Al_2_O_3_ catalysts presented the best catalytic activity among the test samples.

## Discussion

Green diesel (renewable C15-C18 alkanes) can be produced from hydroprocessing of Jatropha oil over Ni-PTA/Al_2_O_3_ catalysts. The maximum conversion of Jatropha oil (98.5 wt%), and selectivity of the C15-C18 fraction (84.5 wt %) was observed at 360 °C, 3.0 MPa, 0.8 h^−1^ using synthetic Ni-PTA/Al_2_O_3_ catalyst (PTA 10 wt.%).

Results of BET and NH_3_-TPD indicated that the pore structure, physicochemical adsorption properties, and acidity of supports and catalysts can affect hydroprocessing catalytic activity. SEM-EDX, TEM and XRD results showed that Ni and W species were distributed on the surface of the synthetic Ni-PTA/Al_2_O_3_ catalyst evenly and no aggregates were observed. This led to the formation of a more active center. XPS clearly showed the existence of assembled PTA on Ni/Al_2_O_3_ catalyst. Ni and W metal oxide/hydroxides not only protected the amorphous structure of the catalyst, aiding in hydrogenation activity, but also enhanced dispersion of active sites.

GC charts showed that C15H32, C16H34, C17H36 and C18H38 were the main products. There was an obvious increase of i-alkane content with increasing PTA-content for all tested catalysts and the amount of smaller molecule alkane increased with increasing reaction temperatures. In this work, the mass balance, through the ratio of the unit time obtained product quantity (7.9 g/h) and consumed reactant quantity (8.1 g/h), is calculated as (7.9/8.1) × 100% = 97.5%. The yields of the main products from hydrotreated Jatropha oil were liquid products at 93.2 wt% (including water 1.5 wt%) and gaseous products at 5.3 wt% over the Ni-PTA(10)/Al_2_O_3_ catalyst at 360 °C, 3.0 MPa, 0.8 h^−1^. The gaseous products contained mainly CO (2.94 wt%), CO_2_ (0.28 wt%), propane (1.57 wt%) and methane (0.48 wt%). The ratio of CO and CO_2_ to H_2_O was calculated as 2.14, in accordance with the ratio of (C15 + C17)/(C16 + C18) in Table 3. From the composition of the gas and liquid products, it can be deduced that the triglyceride is hydrogenated and broken down into various intermediates. Then the intermediates are converted into alkanes by two pathways: Hydrocarbons with the same carbon number as the fatty acids in triglycerides, i.e., C18H38 and C16H34, are the products of hydrodeoxygenation (HDO). Hydrocarbons with one carbon atom less than the fatty acids in triglycerides, i.e., C17H36 and C15H32, are the products of oxygen removal from triglyceride by hydrodecarboxylation (HDC), including decarboxylation + decarbonylation^2^. Figure 9 showed that with increasing reaction temperature, HDC came into play more prominently. Table 3 showed that the relative ratio of (C15 + C17)/(C16 + C18) increased from 1.25 to 4.92 with increasing PTA-content (1–50%). Thus, the selectivity to the decarbonylation plus decarboxylation products (as compared to the hydrodeoxygenation pathway) increased with increasing PTA-content.

Compared with the Ni-PTA/Al_2_O_3_ catalyst using commercial alumina grain and powder as the supports, the synthetic Ni-PTA/Al_2_O_3_ catalyst showed the best catalytic performance. TGA of three catalysts indicated that total amount of coke produced varied with different types of supports. The amount of coke on synthetic Ni-PTA/Al_2_O_3_ catalyst after use for 10 h was lowest. Furthermore, applying transition metal and heteropolyacid catalysts in the hydroprocessing of Jatropha oil can eliminate the sulfurization step and prevent harm from sulfide to the environment and human health.

## Methods

### Catalyst preparation and characterization

In this study, a synthetic Ni-PTA/Al_2_O_3_ catalyst was prepared as follows. First, a synthetic Ni/Al_2_O_3_ (5 wt% Ni) catalyst was prepared with the co-precipitation method. A solution of 70.8 g AlCl_3_·6H_2_O and 4.6 g Ni(NO_3_)_2_·6H_2_O was dissolved in 100 mL of water. An NH_3_·H_2_O solution (20 wt%) was added to the above mixed solution to adjust the pH to 8, with vigorous agitation, and the temperature was maintained at 50 °C for about 2 h. After the reaction was complete, the mixture was aged at 50 °C for 12 h in a water bath. Then the light blue precipitate was collected and washed with water three times. Finally, the precipitate was dried overnight at 100 °C and calcined in a muffle oven at 400 °C for 4 h. Next, the Ni-PTA(x)/Al_2_O_3_ catalyst (where x indicates PTA content from 1 to 50 wt%) was prepared by impregnanting Ni/Al_2_O_3_ catalyst with a solution containing PTA. Impregnated samples were dried at 100 °C for 3 h and calcined at 200 °C for 3 h.

Two commercial alumina supports were selected to compare with the synthetic Al_2_O_3_ support. The Ni-PTA/Al_2_O_3_ catalyst was prepared by wet impregnation, and the detailed preparation was described as follows. The catalyst Ni/Al_2_O_3_ (5 wt%Ni) was prepared by impregnating aqueous solutions of Ni(NO_3_)_2_ on the support Al_2_O_3_ grain (diameter 2–3 mm) or Al_2_O_3_ powder (diameter 0.2–0.3 mm). Impregnated samples were dried overnight at 105 °C and calcined at 400 °C for 4 h. The Ni-PTA/Al_2_O_3_ catalyst was prepared by impregnating the Ni/Al_2_O_3_ catalyst with a solution containing 1 to 50 wt% PTA. Impregnated samples were dried at 100 °C for 3 h and calcined at 200 °C for 3 h.

The BET specific surface area, average pore diameter and pore volume of catalysts were determined by N_2_ isothermal adsorption using the V-Sorb 2800 TP Surface Area and Pore Distribution Analyzer instrument. Scanning electron microscopy (SEM) was conducted with a JSM-6700 (JEOL SEM Co., Japan) instrument to investigate surface morphology and particle size. Elemental mapping images of samples were taken with a CamScan Apollo300 scanning electron microscope (SEM) equipped with X-ray energy-dispersive (EDX) microanalyzer (OXFORD INCA). Transmission electron microscopy (TEM) images were collected from a JEOL JEM-2100F field emission electron microscope operated at 200 kV. The *ex situ* treated samples were supported on Cu grids for the observations. X-ray diffraction (XRD) was used to determine the structural properties of the catalysts. These measurements were performed with a Rigaku D/max-RA XRD analyzer (Cu-Kα) and PIXcel^3D^ detector from the PANalytical Company. Samples were measured in the 2θ range from 10° to 80° (scan speed of 0.02° per second). X-ray photoelectron spectroscopy (XPS) data were obtained using an ESCALab250 electron spectrometer from Thermo Scientific Corporation with nonmonochromatic 300 W AlKα radiation. Pass energy for the narrow scan was 30 eV. Base pressure was at about 6.5 × 10^−10^ mbar. Binding energies were referenced to the C 1s line at 284.8 eV from alkyl or adventious carbon. The acidity of catalysts were measured with a temperature programmed desorption of ammonia (NH_3_-TPD) using a chemisorption analyzer, TP-5080 (Tianjin Xianquan Industrial Trade and Development CO., Ltd) with a thermal conductivity detector. All samples were saturated with NH_3_ at 100 °C, and then flushed with N_2_ to remove the physically adsorbed NH_3_. Finally, desorption of NH_3_ was carried out from 100 to 700 °C at a heating rate of 10 °C/min. Thermo gravimetric analysis (TGA) was performed with a SDT-Q600 analyzer to determine the amount of carbon deposited on the used catalysts. Samples were first heated from 30 to 550 °C at a heating rate of 30 °C/min in N_2_ using a flow of 100 ml/min, at a constant temperature of 550 °C for 15 min. They were then heated linearly at 30 °C/min to 900 °C in 100 ml/min O_2_. The weight loss of samples was processed by microcomputer.

### Catalytic activity measurements

Jatropha oil was purchased from Jiangsu Donghu Bioenergy Co., Ltd. and chemical components were confirmed by GC-MS. The distribution of fatty acids was as follows: palmitoleic acid 0.57%, palmitic acid 12.03%, linoleic acid 33.69%, oleic acid 45.41%, stearic acid 8.14%, eicosenoic acid 0.16%.

Experiments were performed in a fixed-bed reactor equipped with an electrically heating system. A detailed description of the apparatus is provided elsewhere^38^. The non-sulfided catalyst (10 g) was loaded into a stainless steel tubular reactor (1.2 cm I.D and 56 cm in length) and activated *in situ* prior to the experiments with H_2_ at 400 °C and 3.5 MPa for 3 h. The reaction conditions for the catalytic hydroprocessing experiment were as follows: temperature 280–400 °C, pressure 3.0 MPa, LHSV 0.8 h^−1^, and H_2_ to feed ratio of 1000 mL H_2_ gas/mL liquid feed.

The liquid products were withdrawn after stabilization of reaction conditions (6 h) in one-hour intervals and analyzed with off-line gas chromatography (GC) after separation of the water phase. Gaseous products and water were not further analyzed. Liquid products were analyzed using a GC-900C equipped with AT. SE-30 column (L = 30 m, d = 0.32 mm, tf = 0.5 μm) and detected by a flame ionization detector (FID). Helium was used as the carrier gas. The following temperature program was used: initial temperature at 60 °C for 2 min, heating at 15 °C /min to 210 °C, then at 8 °C /min to 270 °C, and at 5 °C /min to 285 °C, with a dwelling time of 5 min at 285 °C. Individual products were identified by GC standards. The conversion of Jatropha oil was calculated as:


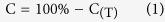


where C_(T)_ is the concentrations of triglycerides (%) in the product oil, determined by GC analysis. The selectivity of C15-C18 alkanes was calculated as:


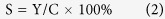


where Y is the yield of the C15-C18 alkane (%), determined by GC analysis, and C is the conversion of Jatropha oil (%), calculated by Eq. (1).

## Additional Information

**How to cite this article**: Liu, J. *et al.* Hydroprocessing of Jatropha Oil for Production of Green Diesel over Non-sulfided Ni-PTA/Al_2_O_3_ Catalyst. *Sci. Rep.* **5**, 11327; doi: 10.1038/srep11327 (2015).

## Supplementary Material

Supplementary Information

## Figures and Tables

**Table 1:** Textural properties of different Al_2_O_3_ samples.

**Samples**	**Specific surface areas, m^2^/g**	**Total pore volume, cm^3^/g**	**Average pore diameter, nm**
Commercial Al_2_O_3_ grain	207	0.38	5.18
Commercial Al_2_O_3_ powder	257	0.42	4.84
Synthetic Al_2_O_3_ support	296	0.43	6.32
Synthetic Ni-PTA/Al_2_O_3_ catalyst	232	0.44	7.49

**Table 2:** Acidity of PTA, synthetic support and catalysts from NH_3_-TPD.

**Surface acidity (mmol/g NH_3_)**	**Weak acidity (100–300 °C)**	**Strong acidity (300–800 °C)**
Pure PTA	0.02	1.71
Synthetic Al_2_O_3_	1.19	0.29
Ni-PTA(10)	0.65	0.67
Ni-PTA(30)	0.53	1.22
Ni-PTA(50)	0.51	1.06

**Table 3:** Hydroprocessing of Jatropha oil on Ni-PTA(1–50)/Al_2_O_3_ catalysts at 360 °C, 3.0 MPa, 0.8 h^−1^.

**Catalyst**	**Ni-PTA(1)**	**Ni-PTA(10)**	**Ni-PTA(30)**	**Ni-PTA(50)**
Conversion (%)	93.32	**98.52**	96.68	95.70
C15-C18 selectivity (%)	78.01	**84.47**	56.31	30.75
(C15 + C17)/(C16 + C18) ratio	1.25	**1.81**	2.06	4.92
Iso/n ratio	0.06	**0.24**	1.03	1.15
Pour point (°C)	20	**−10**	−18	—a

^a^Lower than −20 °C.
