# Supplementary material for: Hydroprocessing of Jatropha Oil for Production of Green Diesel over Non-sulfided Ni-PTA/Al2O3 Catalyst
Source: Sci Rep. 2015 Jul 10;5:11327. doi: 10.1038/srep11327 (PMC4648407; doi:10.1038/srep11327)
Supplement: Supplementary Information [file srep11327-s1.doc]

**Supplementary Data**

**Hydroprocessing of Jatropha Oil for Production of Green Diesel over Non-sulfided Ni-PTA/Al_2_O_3_ Catalyst**

Jing Liu ^1^, Jiandu Lei ^1^,*, Jing He ^1^, Lihong Deng ^1^, Luying Wang ^1^, Kai Fan ^2^, Long Rong ^2^,*

^1^ MOE Key Laboratory of Wooden Material Science and Application, Beijing Forestry University, Beijing 100083, P. R. China

^2^ Key Laboratory for Biomechanics and Mechanobiology of Ministry of Education, School of Biological Science and Medical Engineering, Beihang University, Beijing 100191, P. R. China


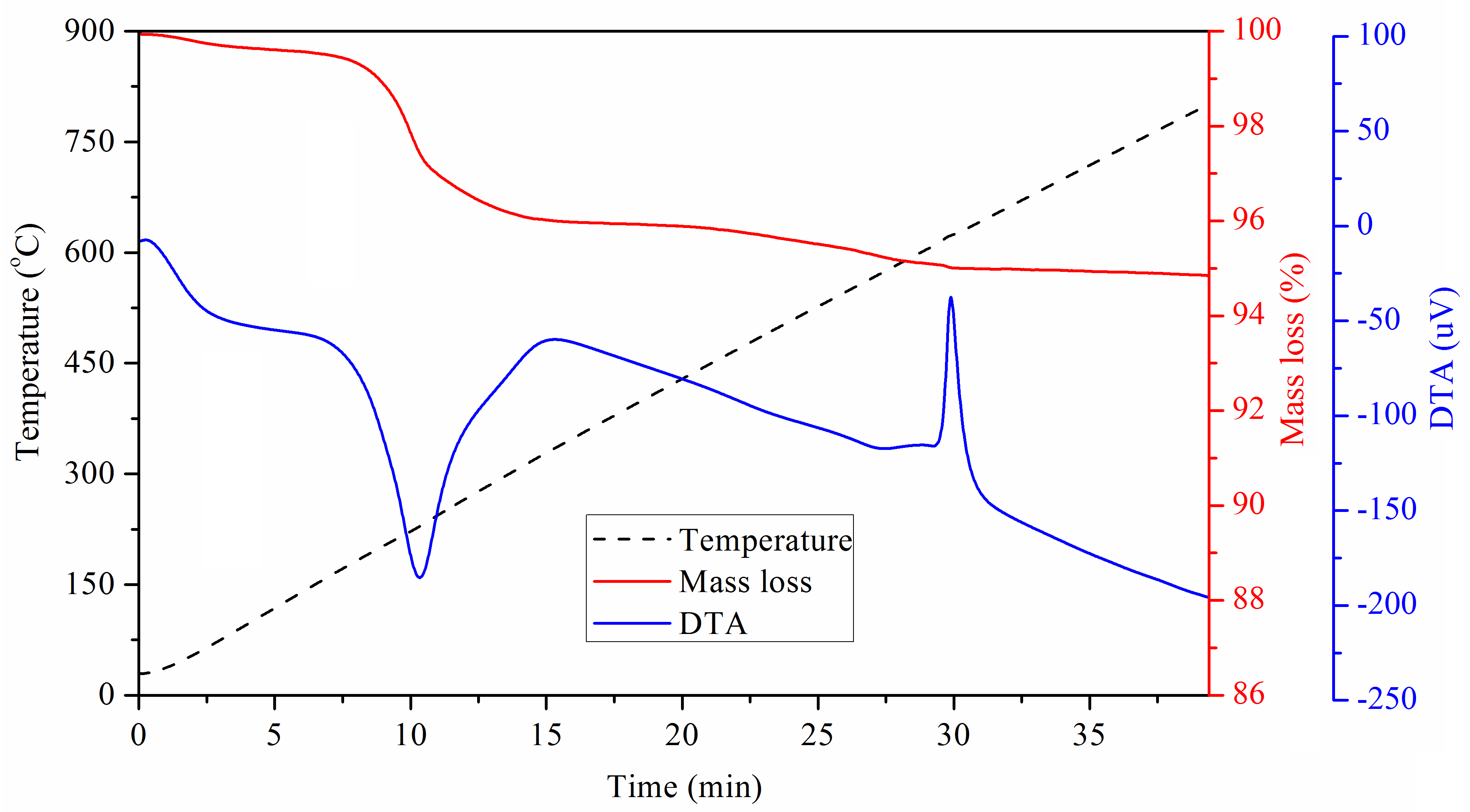


Figure S1 The TG-DTA analysis of PTA (H_3_PW_12_O_40_•6H_2_O). PTA was heated from 30 to 800 °C at a heating rate of 20 °C/min in atmosphere using SHIMADZU, DTG-60/ DSC-60 analyzer.
